# LncRNA TROJAN promotes proliferation and resistance to CDK4/6 inhibitor via CDK2 transcriptional activation in ER+ breast cancer

**DOI:** 10.1186/s12943-020-01210-9

**Published:** 2020-05-11

**Authors:** Xi Jin, Li-Ping Ge, Da-Qiang Li, Zhi-Ming Shao, Gen-Hong Di, Xiao-En Xu, Yi-Zhou Jiang

**Affiliations:** 1 Department of Breast Surgery, Fudan University Shanghai Cancer Center, 270 Dong-An Road, Shanghai, 200032 People’s Republic of China; 2 Cancer Institute, Fudan University Shanghai Cancer Center, 270 Dong-An Road, Shanghai, 200032 People’s Republic of China; 3 Department of Oncology, Shanghai Medical College, Fudan University, 270 Dong-An Road, Shanghai, 200032 People’s Republic of China; 4 Precision Cancer Medicine Center, Fudan University Shanghai Cancer Center, 270 Dong-An Road, Shanghai, 200032 People’s Republic of China; 5 Key Laboratory of Breast Cancer in Shanghai, Shanghai, 200032 China

**Keywords:** Breast cancer, lncRNA TROJAN, CDK4/6 inhibitor

## Abstract

**Background:**

Estrogen receptor-positive (ER+) breast cancers represent approximately two-thirds of all breast cancers and have a sustained risk of late disease recurrence. Cyclin-dependent kinase 4 and 6 (CDK4/6) inhibitors have shown significant efficacy in ER+ breast cancer. However, their effects are still limited by drug resistance. In this study, we aim to explore the role of long noncoding RNA TROJAN in ER+ breast cancer.

**Methods:**

The expression level of TROJAN in breast cancer tissue and cell lines was determined by quantitative real-time PCR. In vitro and in vivo assays as well as patient derived organoid were preformed to explore the phenotype of TROJAN in ER+ breast cancer. The TROJAN-NKRF-CDK2 axis were screened and validated by RNA pull-down, mass spectrometry, RNA immunoprecipitation, microarray, dual-luciferase reporter and chromatin immunoprecipitation assays.

**Results:**

Herein, we showed that TROJAN was highly expressed in ER+ breast cancer. TROJAN promoted cell proliferation and resistance to a CDK4/6 inhibitor and was associated with poor survival in ER+ breast cancer. TROJAN can bind to NKRF and inhibit its interaction with RELA, upregulating the expression of CDK2. The inhibition of TROJAN abolished the activity of CDK2, reversing the resistance to CDK4/6 inhibitor. A TROJAN antisense oligonucleotide sensitized breast cancer cells and organoid to the CDK4/6 inhibitor palbociclib both in vitro and in vivo.

**Conclusions:**

TROJAN promotes ER+ breast cancer proliferation and is a potential target for reversing CDK4/6 inhibitor resistance.

## Background

Breast cancer is the most common malignancy and the second most common cause of cancer death among females [1, 2]. Since the definition of intrinsic molecular subtypes in 2000, breast cancer has been divided into different subtypes and in-depth understood in molecular level [3, 4]. Among those subtypes, estrogen receptor-positive (ER+) breast cancer patients represent approximately two-thirds of all breast cancer patients [5]. ER+ breast cancer patients display better general outcomes than ER−/ERBB2+ or triple-negative patients [6]. However, ER+ breast cancer patients have a high rate of late recurrence, and more than half of all relapse events occur after more than 5 years [7]. Although significant progress has been made in anti-estrogen endocrine therapy, drug resistance remains a problem [8].

Long noncoding RNAs (lncRNAs) have been implicated in various physiological and pathological processes. To date, several lncRNAs have been shown to regulate breast cancer progression. Our previous research discovered that the lncRNA TROJAN is highly expressed in triple-negative breast cancer (TNBC) and contributes to poor outcome by promoting the metastatic potential of TNBC. However, the role of TROJAN in ER+ breast cancer remains unknown.

Cyclin-Dependent Kinase 4 and 6 (CDK4/6) initiate G1/S phase cell cycle entry by phosphorylating and inactivating the retinoblastoma protein Rb1. Recently, the addition of the CDK4/6 inhibitors, such as palbociclib (PD; also known as PD 0332991), significantly improved the progression-free survival of advanced ER+ breast cancer patients [9]. Even though the great majority of patients with advanced disease could benefit from CDK4/6 inhibitors, virtually all will eventually experience progression [10–13]. One of the current therapeutic challenges is to seek more effective drug combinations with CDK4/6 inhibitors to overcome this resistance.

In the current study, we discovered that TROJAN could promote the proliferation of ER+ breast cancer through the regulation of the cell cycle during the G1/S transition. The inhibition of TROJAN using an antisense oligonucleotide (ASO) impaired proliferation. Moreover, the combination of an anti-TROJAN ASO with PD significantly enhanced the efficacy of PD in ER+ breast cancer. Mechanistic investigations showed that TROJAN could bind to NKRF, an NF-κB pathway repressor [14, 15], hindering its interaction with RELA, a transcriptional activator of the NF-κB pathway. Finally, the TROJAN-NKRF complex upregulated the transcription of CDK2 [16, 17], a noncanonical cell cycle S-phase entry pathway that allows tumor cells to escape after CDK4/6 inhibition. These findings provide support for a novel combination of an anti-TROJAN ASO and PD that targets both the CDK4/6 and CDK2 pathways.

## Methods

### Clinical samples

Sixteen paired tumor and paratumor frozen tissues and 92 tumor tissues from ER+ breast cancer patients were immediately collected after surgery at the Department of Breast Surgery at FUSCC (Shanghai, P.R. of China) from 2001 to 2012. The histologic subgroups of these tissues were determined according to their ER and PR expression by immunohistochemistry (IHC). All breast cancer specimens were confirmed to have tumor cell percentages greater than 80%. The survival analysis for the Cancer Genome Atlas (TCGA) cohort of breast cancer was obtained from GEPIA2 (Gene Expression Profiling Interactive Analysis; http://gepia2.cancer-pku.cn/#index).

### RNA isolation and real-time quantitative reverse transcription PCR (qRT-PCR)

Total RNA was isolated from patient specimens or cells using an RNeasy Plus Mini Kit (QIAGEN) following the manufacturer’s protocol. First-strand cDNA synthesis was carried out from total RNA using a PrimeScript RT Reagent Kit with gDNA Eraser (TAKARA). Real-time qRT-PCR was carried out using SYBR Premix Ex Taq (TAKARA) on an ABI 7900HT Fast Real-time PCR system (Applied Biosystems). The results were analyzed with SDS v2.1 software and the 2(−ΔΔC(T)) method.

### Cell lines

MCF7, T47D and embryonic kidney cells (HEK293T) were obtained from the American Type Culture Collection and were cultured under standard conditions. Lentiviruses were generated using the pLKO.1 vector and packaging plasmids (psPAX2 and pMD2.G) in the HEK293FT cell line. The two shRNAs with the best knockdown efficiency were used in subsequent studies (shTROJAN-1: 5′-GCAGTCTCTTAAGCAGATTGA-3′; and shTROJAN-2: 5′-GCAACTGCTGTTAATGAAAGT-3′). PD-resistant MCF7 cells were derived through chronic exposure to 0.1 μmol/L PD for 3 months. Human Tumor Necrosis Factor α (TNFα) was obtained from Cell Signaling Technology.

### Plasmids and cloning procedures

Full-length NKRF, NKRF truncation mutants and full-length CDK2 were introduced into pcDNA3.1(+) (Thermo Fisher Scientific). The pNFκB-luc plasmid was purchased from Beyotime Technology (D2206). The CDK2 promoter was amplified by using a pair of primers (forward: AGGTACCGAGCTCTTACGCGT; and reverse: ACTTAGATCGCAGATCTCGAG) in the MCF7 cell line and was cloned into the pGL3-basic vector (Promega). The luciferase reporter assay was performed by using the Dual-Glo Luciferase Assay System (Promega). All transfections were carried out using Lipofectamine 2000 Reagent (Invitrogen).

### Small interfering RNA (siRNA) delivery

siRNA transfections were carried out using Lipofectamine iMAX Reagent (Invitrogen). The siRNAs for pooled siCDK2 containing the following three individual siRNAs were used: GCACCAAGATCTCAAGAAA, GAGTCCCTGTTCGTACTTA, GGATGTGACCAAGCCAGTA.

### ASO delivery

ASO targeting TROJAN (mAmCmUmUmGTGGCAATAGTmGmAmAmCmA) was purchased from BioSune in Shanghai, China. An ASO that did not correspond to any known human transcripts (Ionis 141,923) was used as the negative control (ASO-Ctrl: mCmCmUmUmCCCTGAAGGTTmCmCmUmCmC).

### In vitro cell growth assay

In vitro cell growth assays were performed by using an IncuCyte system or Cell-counting kit-8 (CCK8) assays. For the IncuCyte system, cells were seeded in 96-well plates and were imaged using the IncuCyte ZOOM system (Essen BioScience). Frames were captured at 12-h intervals in four separate regions per well. Cultures were maintained at 37 °C in an incubator, and the growth rate was analyzed using IncuCyte software (2013A Rev2). For the CCK8 assay, cells were seeded in 96-well plates and were incubated with 10% CCK8 solution at 37 °C for 2 h, and then the absorbance at 450 nm was measured.

### Flow cytometry analysis

Cells were stained with propidium iodide and were analyzed using flow cytometry.

### Xenograft in vivo model

Six-week-old female BALB/c nude mice were used. A 90-day slow-release pellet containing 0.72 mg of 17β-estradiol (Innovative Research of America) was implanted in the back of each mouse. MCF7 cells (5 × 10^6^) were harvested and resuspended in 50 μL of PBS and 50 μL of Matrigel. Cells were injected directly into the mammary fat pads of the mice, and tumor volumes were calculated by V = L×(W × ½)2, where L is the length (longest dimension) and W is the width (shortest dimension). PD was orally administered daily for 28 days at 75 mg/kg. Anti-TROJAN was given by intraperitoneal injection twice per week at 50 mg/kg.

### IHC analysis

Paraffin-embedded tissue sections were deparaffinized at 60 °C for 20 min, cleared in xylene and subjected to a series of graded alcohols. For hematoxylin and eosin (H&E) staining, slides were stained with Mayer’s hematoxylin (Sigma-Aldrich) and 0.1% sodium bicarbonate and were counterstained with Eosin Y solution (Sigma-Aldrich). For IHC, slides were heated with saline-sodium citrate (SSC) buffer at 95–100 °C. After cooling, the slides were blocked with blocking solution (2% goat serum, 2% BSA, and 0.05% Tween in PBS) at room temperature and were incubated with a primary antibody diluted in blocking solution at 4 °C. Endogenous peroxidase activity was quenched with 0.3% H_2_O_2_. Slides were incubated with a horseradish peroxidase (HRP)-conjugated secondary antibody (GeneTech) at room temperature, developed with a 3,3′-diaminobenzidine (DAB) substrate (GeneTech), counterstained with hematoxylin, and dehydrated with a series of graded alcohols. The positive-staining density was measured by a computerized imaging system composed of a Leica charge-coupled device (CCD) DFC420 camera connected to a Leica DM IRE2 microscope (Leica Microsystems Imaging Solutions Ltd). The H-score system was utilized, which evaluated staining intensity (0 to 3) and the percentage of positively stained cells (0 to 100), with a final score ranging from 0 to 300.

### Microarray

Total RNA (300 ng) was amplified using a WT Expression Kit (Ambion) according to the manufacturer’s instructions. cRNA (10 μg) was used for reverse transcription, and 5.5 μg of cDNA was hybridized to a GeneChip® Human Transcriptome Array 2.0 (Affymetrix) according to the manufacturer’s protocol. After overnight hybridization, the chips were terminally labeled and scanned using an Affymetrix GeneChip 3000 7G scanner. The microarray data for TROJAN knockdown in MCF7 cells are available in Gene Expression Omnibus with the accession code GSE148858.

### Colony formation assay

In total, 500 cells were seeded in 6-well plates. Media and inhibitors were replenished every 3 days; after 21 days, cells were stained with crystal violet.

### Patient-derived organoid

The generation of patient-derived organoid was performed as described in a previous study [18]. Briefly, breast cancer tissue was cut into 1–3 mm^3^ pieces and was digested in collagenase (Sigma). The organoid was suspended in basement membrane extract (BME) type 2 (Trevigen) and cultured in 24-well plates. The viability was detected by CCK8 assays.

### Antibodies

The primary antibodies used were anti-Cyclin D1/2 (Proteintech), anti-Cyclin E1/2 (Proteintech), anti-P21/P27 (Proteintech), anti-NKRF (Proteintech or Bethyl), anti-ZMYND8 (Proteintech), anti-ACTB (Proteintech), anti-FLAG (Proteintech), CDK2 (Proteintech), p-CDK2 Thr160 (Cell Signaling Technology), RB1 (Cell Signaling Technology), and p-RB1 S807/811 (Cell Signaling Technology). The HRP-linked anti-mouse (1/5000) and anti-rabbit antibodies (1/5000) were purchased from Cell Signaling Technology.

### Primers

TROJAN-F: GGTTCTGGAAATTCTACTTGTGGA; TROJAN-R: AAACTGTTTTATGTGACATGCATCA; U6-F: CTCGCTTCGGCAGCACA; U6-R: AACGCTTCACGAATTTGCGT; CDK2-F: CAGAGCTTTTGGAGTCCCTGT; CDK2-R: AAAGATCCGGAAGAGCTGGT.

### Stable isotope labeling with amino acids in cell culture (SILAC) preparation

Dulbecco’s Modified Eagle’s Medium (DMEM) medium (Thermo Scientific) was supplemented with 10% dialyzed fetal bovine serum (FBS) (Thermo Scientific) and 1% streptomycin/penicillin. SILAC medium containing 42 mg/L lysine, 73 mg/L arginine and 200 mg/L proline was added to prevent the arginine to proline conversion. The heavy culture medium was supplemented with lysine (U-13C6, 99%; U-15 N2, 99%) (Lys8) and arginine (U-13C6, 99%; U-15 N4, 99%) (Arg10). The culture medium was supplemented with lysine (4,4,5,5-D4, 96–98%) (Lys4) and arginine (U-13C6, 99%) (Arg6). All amino acid isotypes were synthesized by Cambridge Isotope Laboratories, and unlabeled amino acids were purchased from Sigma-Aldrich. Cells were grown in parallel for at least five generations.

### RNA pull-down assay and mass spectrometry

The RNA pull-down assay was performed as previously described [19]. Briefly, full-length sense and antisense TROJAN sequences were cloned into pGEM-T Easy (Promega). In vitro transcription was carried out using a HiScribe™ T7 Quick High Yield RNA Synthesis Kit (New England Biolabs), and RNA was purified using an RNeasy MinElute Cleanup kit (QIAGEN). TROJAN was labeled using a Biotin 3′ End DNA Labeling Kit (Thermo Scientific). SILAC-labeled cells were harvested and resuspended in freshly prepared lysis buffer (50 mM Tris, pH 7.4, 150 mM NaCl, 1% NP-40, an 0.25% sodium deoxycholate) supplemented with 50 U/mL RNaseOUT recombinant ribonuclease inhibitor (Thermo Scientific), 50 U/mL SUPERaseIn RNase inhibitor (Thermo Scientific) and a protease/phosphatase inhibitor cocktail (Roche). In total, 10 mg of SILAC-labeled cell lysates was harvested using 0.1 μg/μL yeast transfer RNA (tRNA) and 5 mM MgCl2. Sense and antisense RNAs were captured on magnetic beads (Pierce) and were incubated with Lys0 & Arg0, Lys4 & Arg6 and Lys8 & Arg10 cell lysates in protein-RNA-binding buffer (Thermo Scientific) overnight at 4 °C with agitation. RNA-binding protein complexes were washed five times with ice-cold wash buffer and were boiled in SDS lysis buffer.

Liquid chromatography-tandem mass spectrometry (LC-MS/MS) experiments were performed on a high performance liquid chromatography (HPLC) system comprised of a nanoACQUITY Binary Solvent Manager LC pump and a nanoACQUITY Sample Manager (all from Waters Corporation, Milford, MA) connected to an LTQ-Orbitrap XL mass spectrometer (Thermo Fisher Scientific). Samples were injected onto an Acclaim PepMap precolumn (0.1 × 20 mm, Thermo Fisher Scientific) for 2 min at a flow rate of 8 μL/min and were subsequently separated on an Acclaim PepMap rapid separation liquid chromatography (RSLC) column (0.075 × 250 mm, Thermo Fisher Scientific) at a flow rate of 300 nL/min. The mobile phases were 0.1% formic acid (phase A and the loading phase) and 99.9% acetonitrile with 0.1% formic acid (phase B). A 90-min linear phase B gradient from 3 to 35% was employed. The separated samples were introduced into the mass spectrometer via a nanoelectrospray source (Thermo Electron Corporation). The spray voltage and heating capillary were set at 1.6 kV and 200 °C, respectively. The mass spectrometer was operated in the data-dependent mode, and each duty cycle consisted of one full MS survey scan in the 350 ~ 1600 Da mass range with a resolution power of 60,000 using the Orbitrap section, followed by MS2 experiments to identify the 10 strongest peaks with the linear trap quadruple (LTQ) section. Peptides were fragmented in the LTQ section using collision-induced dissociation with helium. Normalized collision energy values were set at 35%, and previously fragmented peptides were excluded for 60 s.

Protein searches were performed with MaxQuant 1.5.3.8 software against a database containing 246 common contaminant proteins and the SWISSPROT database for humans (version 2014_07, downloaded from ftp://www.uniprot.org). Triple multiplicities for Lys0 & Arg0, Lys4 & Arg6 and Lys8 & Arg10 were used for the SILAC quantitation, and the variable modifications were methionine (M) oxidation, protein N-terminal acetylation and lysine (K) acetylation. The maximum number of missed cleavage sites was set to 2, and the false discovery rate (FDR) identification acceptance criteria was less than 1% for peptides, proteins and modification sites.

### RNA immunoprecipitation (RIP)

RIP was performed using a Magna RIP Kit according to the manufacturer’s instructions (Millipore). Briefly, 2 × 10^7^ cells were trypsinized and rinsed twice with ice-cold PBS, resuspended in an equal pellet volume of RIP lysis buffer supplemented with a protease inhibitor cocktail and an RNase inhibitor and subjected to a single freeze-thaw cycle to gently lyse the cells. A total of 5 μg of antibody was added to the magnetic beads, and the mixture was incubated for 30 min at room temperature in RIP wash buffer with agitation. The beads were washed three times in RIP wash buffer, and the cell lysates were added and incubated at 4 °C overnight. Each immunoprecipitant was resuspended in proteinase K buffer (1.2 μg/μL proteinase K and 1% SDS) and was incubated at 55 °C for 30 min. RNA was isolated by phenol, chloroform and isoamyl alcohol according to the manufacturer’s instructions and was detected by qRT-PCR.

### Western blot analysis

Cells were lysed in SDS lysis buffer (50 mM Tris (pH 8.1), 1 mM EDTA, 1% SDS, 1 mM fresh dithiothreitol, sodium fluoride and leupeptin). The lysates were centrifuged at 10,000 g for 20 min, supernatants were collected, and protein concentrations were determined with a bicinchoninic (BCA) protein assay kit (Pierce). A total of 20–60 μg of protein was separated by SDS-PAGE and transferred to PVDF membranes (Millipore). The primary and secondary antibodies were described above, and a SuperSignal™ West Femto Substrate Trial Kit (Pierce) was used as the enhanced chemiluminescence substrate. ImageJ was used to quantify the Western blotting results by densitometry.

### RNA in situ hybridization (ISH)

For fluorescent ISH, cells were rinsed in PBS twice, fixed in 4% formaldehyde in PBS (pH 7.4) for 30 min at room temperature, incubated in a H_2_O_2_ solution for 10 min and digested in protease solution for 10 min. The slides were then hybridized with a custom probe, Hs-LOC105372310-O2, in a HybEZ oven (Advanced Cell Diagnostics) at 40 °C for 2 h. After signal amplification, the slides were conjugated with TSA® Plus Cy3 (PerkinElmer). The slides were then incubated with anti-NKRF antibody overnight at 4 °C. After incubation with the fluorescein-conjugated secondary antibody, the slides were mounted with ProLong® Gold Antifade Reagent with DAPI.

### Immunoprecipitation assay

In total, 1 × 10^7^ cells were lysed in lysis buffer (50 mM Tris, pH 7.4, 150 mM NaCl, 1% NP-40, and 0.25% sodium deoxycholate) and were immunoprecipitated with anti-FLAG M2 magnetic beads at 4 °C overnight. The immunoprecipitants were thoroughly washed with lysis buffer five times and were eluted by boiling with SDS loading buffer for 5 min.

### Chromatin immunoprecipitation (ChIP)

Briefly, 1 × 10^7^ cells were crosslinked with 1% formaldehyde, sonicated to create 200–500 bp fragments in ChIP lysis buffer (50 mM HEPES, pH 7.5, 500 mM NaCl, 1 mM EDTA, 1% Triton, and 0.1% Na-deoxycholate, supplemented with a protease inhibitor cocktail), and incubated with 3 μg of specific antibodies with protein A/G magnetic beads (Millipore). The beads were washed 3 times with lysis buffer, 3 times with wash buffer (50 mM HEPES, 300 mM LiCl, 1 mM EDTA, 0.5% NP-40, and 0.7% Na-deoxycholate), and once with TE. Each immunoprecipitant was eluted and reverse cross-linked in elution buffer (50 mM Tris-HCl, pH 8.0, 10 mM EDTA, and 1% SDS) at 65 °C for 6 h. After RNase A and Proteinase K digestion, DNA samples were isolated with phenol, chloroform and isoamyl alcohol according to the manufacturer’s instructions and were analyzed by qRT-PCR.

### Ethical approval

All the procedures involving patients were performed in accordance with the Declaration of Helsinki (1964, amended in 1975, 1983, 1989, 1996, and 2000) of the World Medical Association. This study was approved by the Ethics Committee of FUSCC, and each participant signed an informed consent document. The animal protocols were approved by the Animal Welfare Committee of Shanghai Medical College at Fudan University.

### Statistical analysis

Statistical analysis was performed using SPSS and R software. *P* < 0.05 was considered significant. The survival curves were constructed with the Kaplan-Meier method and were compared with the log-rank test. Relapse-free survival (RFS) was assessed from the date of surgery to the date of local relapse or distant relapse. Overall survival was assessed from the date of surgery to the date of death or the last follow-up. Patients without events or death were censored at the last follow-up.

## Results

### TROJAN was highly expressed and predicted poor overall survival in ER+ breast cancer

**Fig. 1:**
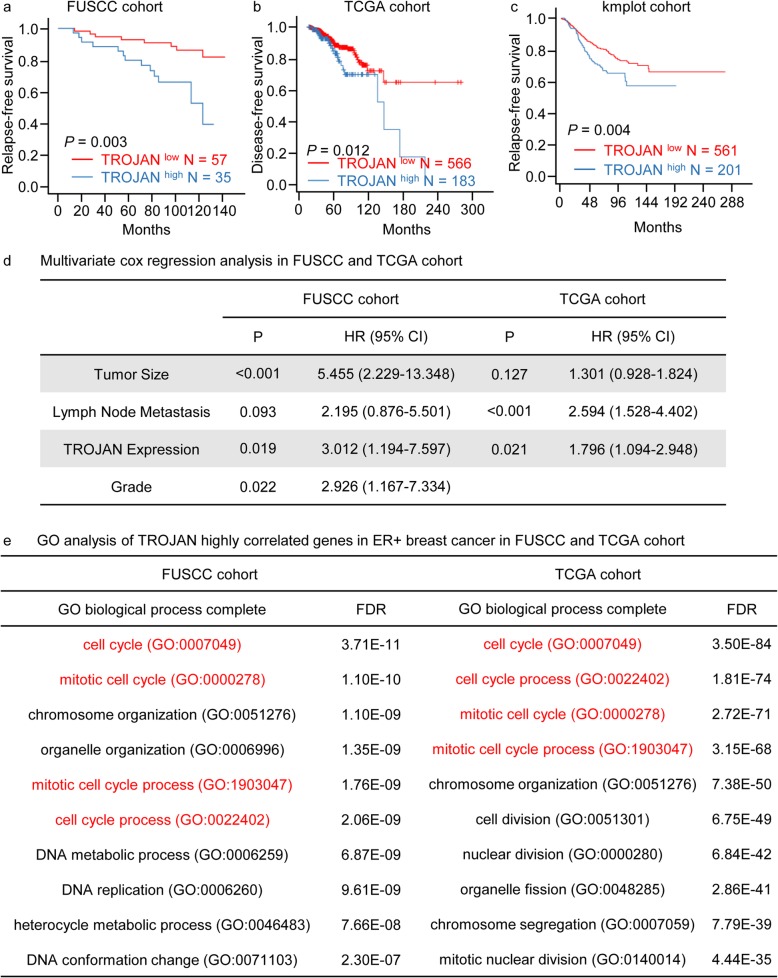
TROJAN was highly expressed and predicted poor survival in ER+ breast cancer. **a** Kaplan-Meier analysis of the relapse-free survival of 92 ER+ breast cancer patients in the FUSCC cohort. A log-rank test was used to determine the statistical significance between the low TROJAN expression group (*n* = 57) and the high TROJAN expression group (*n* = 35). **b** Kaplan-Meier analysis of the disease-free survival of 600 ER+ breast cancer patients in the TCGA cohort (low TROJAN expression group, *n* = 566; high TROJAN expression group, *n* = 183). **c** Kaplan-Meier analysis of the relapse-free survival of 762 ER+ breast cancer patients in the kmplot cohort (low TROJAN expression group, *n* = 561; high TROJAN expression group, *n* = 201). **d** Multivariate cox regression of clinicopathologic variables and TROJAN expression levels in the FUSCC cohort and TCGA cohort. HR, hazard ratio; CI, confidence interval. **e** Gene Ontology (GO) analysis of the top 250 genes correlated with TROJAN in FUSCC ER+ breast cancer patient cohort (GSE115144) and TCGA cohort. FDR, false discovery rate

We first used qRT-PCR to detect the expression of TROJAN in 16 paired ER+ breast cancer and normal breast tissue samples. The expression of TROJAN was significantly higher in tumor tissue than in paratumor normal tissue (Additional files 1, Fig. S1a). We used qRT-PCR to measure the expression of TROJAN in another ER+ breast cancer cohort that included survival information. With an optimal cutoff point determined by X-tile statistical software, we confirmed that TROJAN was associated with reduced relapse-free survival (RFS) among ER+ breast cancer patients (Fig. 1a). In TCGA database, TROJAN (we used the ENST00000624228.1 transcript as a substitution in GEPIA2) was associated with reduced disease-free survival in ER+ breast cancer patients (Fig. 1b). Similarly, in kmplot.com [20], TROJAN (we used the 242346_x_at probe set as a substitution) was also associated with reduced disease-free survival in ER+ breast cancer patients (Fig. 1c). We included tumor size, lymph node metastasis, grade (only included in FUSCC cohort) and TROJAN expression in multivariate cox regression analysis in FUSCC and TCGA cohort (Fig. 1d). We determined that TROJAN was an independent prognostic factor. We selected the 250 genes which were most highly positively correlated with TROJAN in FUSCC ER+ breast cancer patient cohort (GSE115144) and TCGA cohort to perform Gene Ontology (GO) analysis, which demonstrated that there was a greater enrichment of cell cycle-related biological processes than other processes (Fig. 1e). Using the GTEx database (https://www.gtexportal.org/), we found that the median expression of TROJAN is relatively low in normal tissue (Additional files 1: Fig. S1b). We detected the expression of TROJAN in different cell lines, and we found that TROJAN was highly expressed in the ER+ breast cancer cell lines (MCF7, BT474, and T47D) compared with two normal cell lines (HMEC and MCF10A human mammary epithelial cells; Additional files 1: Fig. S1c). Thus, we concluded that TROJAN might promote the progression of ER+ breast cancer via the regulation of the cell cycle.

### Anti-TROJAN ASO therapy sensitized ER+ breast cancer cells to PD.

**Fig. 2:**
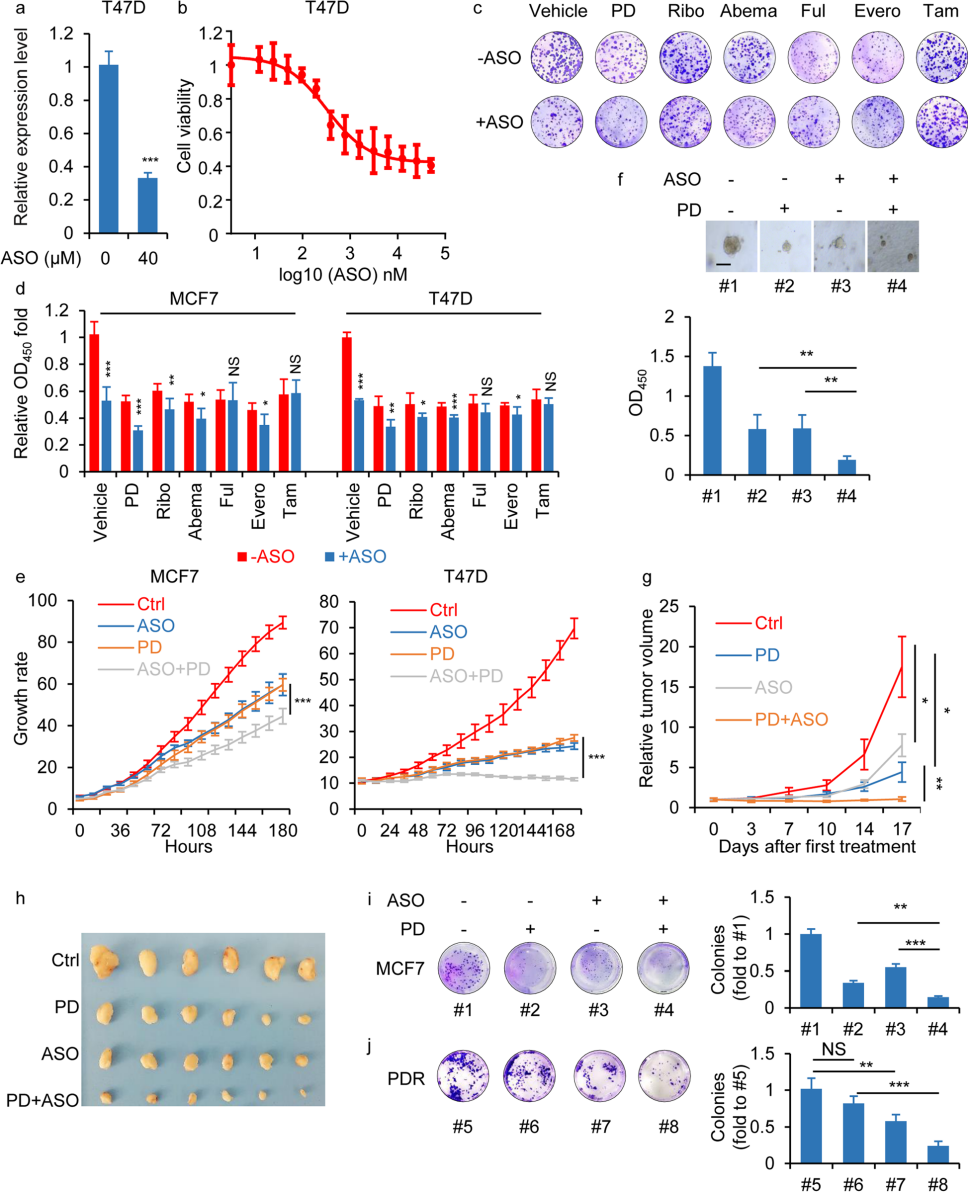
Anti-TROJAN ASO therapy sensitized ER+ breast cancer cells to PD. **a** In vitro free-uptake assay in T47D cells. Cells were treated by 0 or 40 μM of the ASO. The qRT-PCR detection of TROJAN expression. *n* = 3 independent experiments. Unpaired t test. **b** Cell viability of T47D treated with different concentration of ASO. *n* = 3 independent experiments. **c** Clonogenic survival assays of MCF7 cells treated with ASO in combination with palbociclib (PD), ribociclib (Ribo), abemaciclib (Abema), fulvestrant (Ful), everolimus (Evero) or tamoxifen (Tam). *n* = 3 independent experiments. **d** In vitro growth assay of MCF7 and T47D cells treated with ASO in combination with palbociclib (PD), ribociclib (Ribo), abemaciclib (Abema), fulvestrant (Ful), everolimus (Evero) or tamoxifen (Tam). *n* = 3 independent experiments. Unpaired t test. **e** In vitro growth assays of MCF7 and T47D cells that were treated with 0.1 μM PD or/and 40 μM anti-TROJAN ASO. *n* = 3 independent experiments. Two-way ANOVA analysis was used. **f** One ER+ breast cancer patient-derived organoid treated with 0.1 μM PD or/and 40 μM anti-TROJAN ASO. The viability as measured by OD_450_ is shown. Scale bar: 100 μm. *n* = 3 independent experiments. One-way ANOVA analysis was used. **g, h** In vivo growth of MCF7 cells (mean ± standard error of mean; *n* = 6) after 17 days of treatment with PD and anti-TROJAN ASO, individually or in combination. Tumor volume quantification **g** and representative tumor images **h** are shown. Each row in **h** represents a treatment. Columns represent repetitions. Two-way ANOVA analysis was used. **i, j** Clonogenic survival assays in MCF7 cells **i** and PDR cells **j** treated with 0.1 μM PD or/and 40 μM anti-TROJAN ASO. The number of colonies is shown. *n* = 3 independent experiments. One-way ANOVA analysis was used. *p < 0.05, **p < 0.01, ***p < 0.001 and NS: not significant

Next, we used an ASO to evaluate the therapeutic potential of TROJAN in ER+ breast cancer. Compared to T47D control cells, T47D cells transfected with the ASO exhibited significantly reduced TROJAN expression as well as reduced proliferation ability (Fig. 2a-b). Next, we assessed the combination regimen of ASO with the several classic inhibitors (palbociclib, ribociclib, abemaciclib, fulvestrant, everolimus and tamoxifen) for ER+ breast cancer patients. Through the colony formation assay and the proliferation assay, we found that the combination of ASO and CDK4/6 inhibitors (palbociclib, ribociclib and abemaciclib) could further reduce the growth of cells than those inhibitors alone (Fig. 2c-d). Further, we assessed the efficacy of ASO-TROJAN, PD or the combination of these two treatments. An in vitro proliferation assay showed that the growth rate of MCF7 and T47D cells was moderately (but significantly) inhibited by treatment with a single agent (ASO-TROJAN or PD) and was further inhibited (also significantly) by a combination of ASO-TROJAN and PD (Fig. 2e). Using the organoid derived from one ER+ breast cancer patient, we also found that the viability of the organoid was further reduced by the combination of ASO-TROJAN and PD compared to that in response to each single agent (Fig. 2f). In mammary fat pad xenografts, both CDK4/6 and TROJAN inhibition exhibited significant tumor growth reduction, whereas the combination of CDK4/6 and TROJAN inhibition further reduced tumor growth (Fig. 2g-h). We also generated MCF7 cells with acquired resistance to PD (PDR). The half-maximal inhibitory concentration (IC50) of palbociclib, ribociclib and abemaciclib were all increased in PDR cells (Additional files 2: Fig. S2a). The phosphorylation of RB1 under the exposure in palbociclib, ribociclib and abemaciclib was also increased (Additional files 2: Fig. S2b). Using a colony formation assay, we observed that the colony number was further reduced by the combination of ASO-TROJAN/PD compared to that with each single agent (Fig. 2i). In PDR cells, PD treatment alone did not affect the clonogenic ability while the ASO-TROJAN alone could partially decrease the clonogenic ability (Fig. 2j). Interestingly, the combination treatment of ASO-TROJAN/PD could significantly decrease the clonogenic ability compared to PD treatment cells (Fig. 2j). Taken together, these data indicated that the anticancer efficacy of the blockade of CDK4/6 could be significantly enhanced when it was combined with TROJAN inhibition.

### Downregulation of TROJAN inhibited ER+ breast cancer proliferation by inducing G1/S cell cycle arrest

**Fig. 3:**
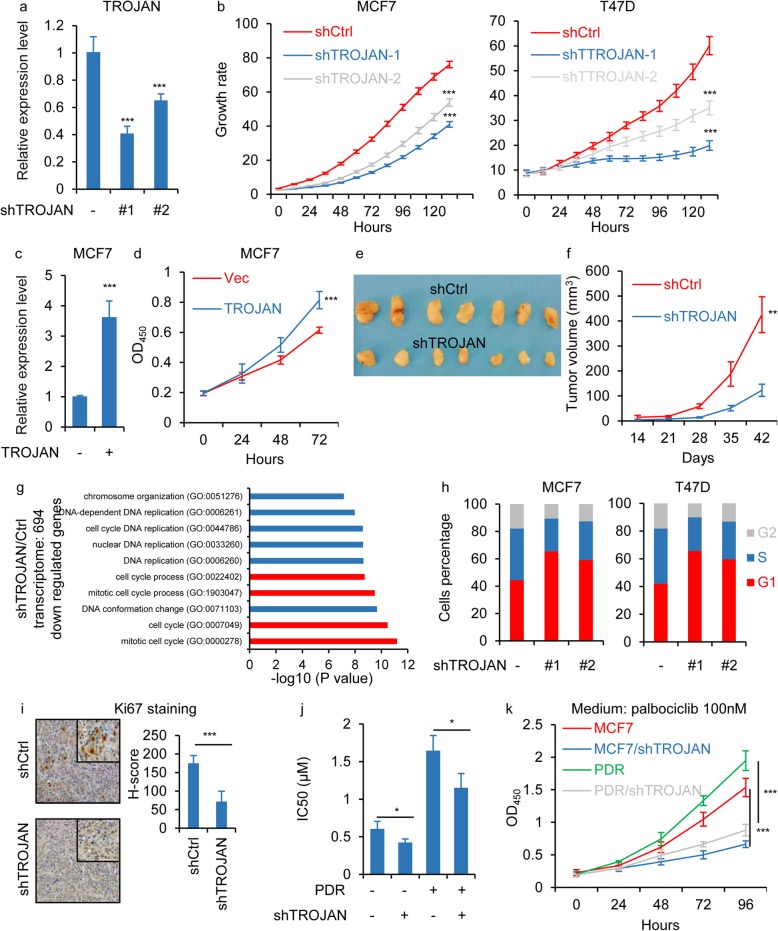
TROJAN promoted cell proliferation through the regulation of G1/S cell cycle entry. **a** qRT-PCR analysis TROJAN transcription in MCF7 cells expressing control (Ctrl) or TROJAN short-hairpin RNAs (shRNAs). *n* = 3 independent experiments. Unpaired t test was used. **b** In vitro growth curves of MCF7 and T47D cells expressing Ctrl or TROJAN shRNAs. *n* = 3 independent experiments. Two-way ANOVA analysis was used. **c** qRT-PCR detection of TROJAN transcription in MCF7 cells overexpressing a control vector (Vec) or TROJAN. *n* = 3 independent experiments. Unpaired t test was used. **d** In vitro growth curves of MCF7 cells expressing Vec or TROJAN. *n* = 3 independent experiments. Two-way ANOVA analysis was used. **e, f** In vivo growth of MCF7 cells (42 days; mean ± standard error of mean; *n* = 7) expressing Ctrl or TROJAN shRNA. Tumor volume quantification and representative tumor images are shown. Two-way ANOVA analysis was used. **g** Pathway analysis of 694 down regulated genes after TROJAN knockdown identified by a microarray. Top 10 pathways according to -log10 (*P* value) are shown. **h** Flow cytometry analysis showing the constituent ratios of different cell cycle phases in the TROJAN knockdown MCF7 and T47D cell lines. *n* = 3 independent experiments. **i** Immunohistochemical detection of Ki67 in a mammary fat pad xenograft model generated by injecting MCF7 cells with TROJAN knockdown. The H-score is shown. Unpaired t test was used. **j** IC50 values of PD in MCF7, MCF7 with TROJAN knockdown, PD resistance MCF7 (PDR) and PDR with TROJAN knockdown cells. *n* = 3 independent experiments. One-way ANOVA analysis was used. **k** In vitro growth assay of MCF7, MCF7 with TROJAN knockdown, PD resistance MCF7 (PDR) and PDR with TROJAN knockdown cells treated with 0.1 μM PD.* n* = 3 independent experiments. Two-way ANOVA analysis was used. *p < 0.05, **p < 0.01 and ***p < 0.001

As TROJAN might regulate the cell cycle in ER+ breast cancer, we performed an in vitro proliferation assay in TROJAN knockdown cells and found that the proliferative potential of MCF7 and T47D cells was impaired (Fig. 3a-b) with TROJAN knockdown. With the overexpression of TROJAN, the proliferative potential of MCF7 was promoted (Fig. 3c-d). We used a mammary fat pad injection model in female nude mice to investigate the effect of TROJAN on ER+ breast cancer proliferation in vivo. The downregulation of TROJAN significantly reduced the tumor volume (Fig. 3e-f). To examine the regulatory role of TROJAN by analyzing comprehensive gene expression data, we performed transcriptional gene microarray analysis using both MCF7 TROJAN-down regulated cells and MCF7 control cells. The GO analysis of 694 down regulated genes in TROJAN-downregulated cells revealed that several cell cycle-related biological processes were significantly altered (Fig. 3g). Such result was not observed in the up regulated genes in TROJAN-down regulated cells (Additional files 3: Fig. S3a). With further research using flow cytometry, we determined that the downregulation of TROJAN resulted in an increase in G1 phase cells (Fig. 3h). We detected a serious G1/S cell cycle markers by western blot and confirmed that Cyclin E1/2 were decreased, P21/P27 was increased by TROJAN knockdown (Additional files 3: Fig. S3b). We also observed that the proliferation marker MKI67 was positively stained less frequently in TROJAN knockdown tumor samples than in control tumor samples (Fig. 3i). The IC50 was decreased in TROJAN knockdown MCF7 and PDR cells compared to that in control MCF7 and PDR cells (Fig. 3j). In medium containing PD, the growth rate of PDR cells was higher than MCF7 cells and TROJAN knockdown PDR cells (Fig. 3k). Together, these results demonstrate that TROJAN promotes ER+ breast cancer progression through the regulation of G1/S cell cycle arrest.

### TROJAN associated with the NKRF protein in ER+ breast cancer

**Fig. 4:**
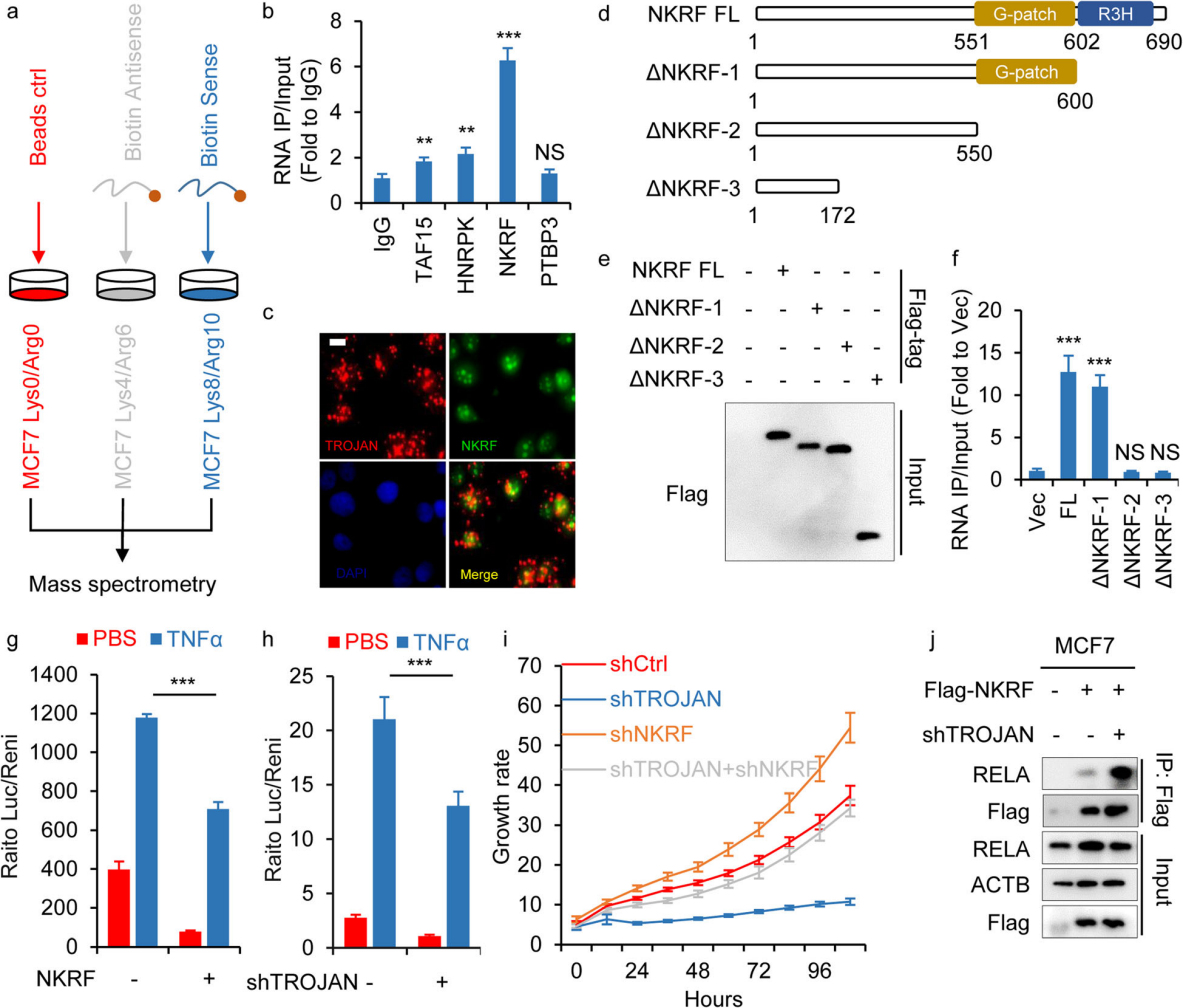
TROJAN associated with the NKRF protein in ER+ breast cancer. **a** Schematic diagram of the RNA pull-down assay combined with quantitative proteomics. **b** RIP assay and the subsequent qRT-PCR assay. The relative quantification of TROJAN in MCF7 RNA-protein complexes immunoprecipitated with IgG or antibodies against four potential interacting proteins (TAF15, HNRPK, NKRF and PTBP3). *n* = 3 independent experiments. **c** Confocal RNA fluorescence ISH and immunofluorescence images showing the colocalization of TROJAN and NKRF in MCF7 cells. Scale bar: 10 μm. *n* = 3 independent experiments. **d** Schematic representation of Flag-tagged full-length human NKRF and NKRF deletion mutants. **e** The anti-Flag Western blot image showing NKRF full-length or deletion mutant expression in HEK293T cells. **f** RIP assay and the subsequent qRT-PCR assay performed in HEK293T cells ectopically expressing the control vector (Vec), full-length Flag-tagged ZMYND8 and ZMYND8 deletion mutants. The relative quantification of TROJAN expression in RNA-protein complexes immunoprecipitated with a Flag antibody. *n* = 3 independent experiments. **g, h** Luciferase reporter assay detecting the activity of the NF-κB pathway during NKRF over expression or TROJAN knockdown with or without TNFα treatment (20 ng/mL for 6 h). *n* = 3 independent experiments. **i** In vitro growth of MCF7 cells expressing TROJAN and/or NKRF shRNA. *n* = 3 independent experiments. **j** Western blot images showing the interaction of RELA with NKRF during TROJAN knockdown. *n* = 3 independent experiments. Unpaired t test, **p < 0.01, ***p < 0.001 and NS: not significant

To further explore the mechanism underlying the phenotype of TROJAN in ER+ breast cancer, we performed RNA pull-down assays combined with stable isotope labeling with amino acids in cell culture SILAC-based quantitative proteomics to identify proteins associated with TROJAN. Full-length sense and antisense TROJAN sequences were transcribed in vitro, purified and labeled with biotin at their 3′ ends. TROJAN sense sequences, TROJAN antisense sequences and blank beads were incubated with MCF7 cell lysates labeled with the stable isotopes of Lys8 & Arg10, Lys4 & Arg6 and Lys0 & Arg0 (Fig. 4a). After excluding general RNA-binding proteins, four potential tumor progression-related proteins (TAF15, HNRPK, NKRF and PTBP3) were identified (Additional files 4: Fig. S4a). We next performed RIP to screen the key protein interactions. Compared with immunoglobulin G (IgG), TAF15, HNRPK, and NKRF were significantly enriched for TROJAN, and the enrichment fold change in NKRF was the highest (Fig. 4b). Finally, we focused on NKRF as a key TROJAN-interacting protein in ER+ breast cancer. The confocal microscopy of TROJAN fluorescence ISH and NKRF immunofluorescence showed the colocalization of TROJAN and NKRF in the nucleus (Fig. 4c). To identify the binding regions between TROJAN and NKRF, we first ectopically expressed full-length Flag-NKRF as well as three mutants: the truncated R3H domain (ΔNKRF-1) mutant, the truncated R3H + G-patch domain (ΔNKRF-2) mutant, and the NKRF N-terminal (ΔNKRF-3) mutant (Fig. 4d, e). Using a RIP assay, we demonstrated that deleting the R3H + G-patch domain, but not deleting only the R3H domain, could abolish the TROJAN-NKRF interaction (Fig. 4f). As the G-patch domain was already known to have an RNA-binding function [21], we concluded that TROJAN could bind to the G-patch domain of NKRF.

The NKRF protein was reported to suppress the NF-κB pathway [15]. A luciferase reporter assay using an NF-κB-responsive promoter revealed that NKRF over expressed or TROJAN down regulated cells had decreased luciferase activity after TNFα treatment (Fig. 4g-h) compared to control cells. The knockdown of NKRF promoted the proliferation of MCF7 cells (Additional files 3: Fig. S3b-c). These results, together with previous research [14], indicated that NKRF could negatively regulate tumor growth. Next, we performed an in vitro proliferation assay in MCF7 cells expressing TROJAN and/or NKRF shRNAs. We found that the inhibition of proliferation by TROJAN knockdown was weakened by the additional knockdown of NKRF, which indicated that the TROJAN knockdown-induced phenotype could be partially rescued by NKRF (Fig. 4i). Using a co-immunoprecipitation assay, we observed that the binding capacity between NKRF and RELA was enhanced after the knockdown of TROJAN (Fig. 4j), suggesting that TROJAN could hinder the interaction between NKRF and RELA. According to our previous report [19], ZMYND8 interacted with TROJAN in TNBC. We knocked down ZMYND8 in the MCF7 cell line and confirmed that the proliferation was increased (Additional files 3: Fig. S3d-e) compared to that in the controls. However, ZMYND8 could not rescue the proliferation inhibition caused by TROJAN knockdown in MCF7 cells, indicating that TROJAN did not participate in the ZMYND8-mediated pathway in ER+ breast cancer (Additional files 3: Fig. S3e). Collectively, these data indicate that TROJAN could bind to NKRF and hinder its interaction with RELA.

### TROJAN regulated the transcription of CDK2 via NKRF

**Fig. 5:**
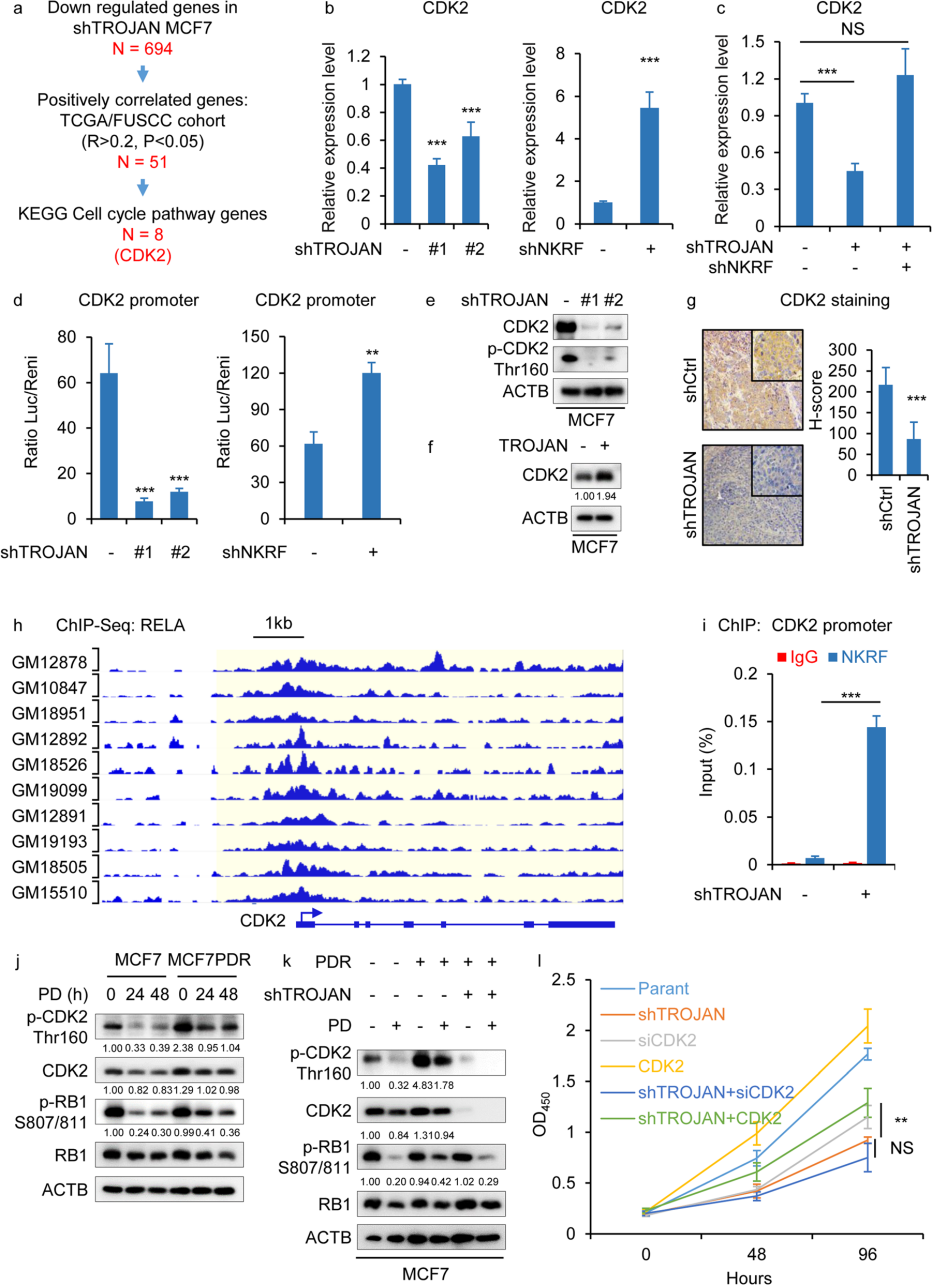
TROJAN promoted the transcription of CDK2 via NKRF. **a** Schematic diagram of the screening of the eight potential target genes (CCNA2, MCM7, CDC45, CDK2, MCM3, PCNA, BUB1 and MCM6). First, 694 genes down regulated by TROJAN knockdown (according to transcriptome analysis of TROJAN knockdown MCF7 cells) were used for screening. After the overlapping with pearson correlation analysis of genes that were positively correlated with TROJAN (R > 0.2, *P* < 0.05) in FUSCC and TCGA cohort, as well as genes from the KEGG cell cycle pathways, the potential eight target genes were identified. **b** qRT-PCR detecting the expression of CDK2 during TROJAN or NKRF knockdown. *n* = 3 independent experiments. Unpaired t test was used. **c** qRT-PCR detecting the expression of CDK2 in MCF7 cells with TROJAN or TROJAN/NKRF knockdown. *n* = 3 independent experiments. Unpaired t test was used. **d** Luciferase reporter assay detecting the activity of the CDK2 promoter during TROJAN or NKRF knockdown. *n* = 3 independent experiments. Unpaired t test was used. **e** Western blot images of p-CDK2 Thr160 and total CDK2 in MCF7 cells expressing TROJAN shRNAs. *n* = 3 independent experiments. **f** Western blot images of CDK2 in MCF7 cells ectopically expressing TROJAN. *n* = 3 independent experiments. **g** Immunohistochemical detection of CDK2 in a mammary fat pad xenograft model generated by injecting MCF7 cells with TROJAN knockdown. The H-score is shown. **h** RELA ChIP-Seq signals in lymphocyte at CDK2 nearby genomic location. **i** qRT-PCR analysis of NKRF at the CDK2 promoter after ChIP assays in MCF7 cells expressing control or TROJAN shRNA. *n* = 3 independent experiments. Unpaired t test was used. **j** Western blot images of MCF7 and PDR cells treated for 24 and 48 h with 0.1 μM PD and blotted for phospho-CDK2 (p-CDK2) Thr160, total CDK2, phospho-RB1 (p-RB1) S807/811, and total RB1. *n* = 3 independent experiments. **k** Western blot images of MCF7, PDR and PDR with TROJAN knockdown cells treated for 24 h with 0.1 μM PD and blotted for p-CDK2 Thr160, total CDK2, p-RB1 S807/811, and total RB1. *n* = 3 independent experiments. **l** In vitro growth curves of MCF7 cells with ± CDK2 knockdown, ± TROJAN knockdown and ± CDK2 over expression. Two-way ANOVA analysis was used. **p < 0.01, ***p < 0.001 and NS: not significant

To investigate the downstream targets regulated by TROJAN, we reanalyzed the microarray data from MCF7 cells with TROJAN knockdown and from 21 ER+ breast cancer patients published previously (GSE115144) [22]. In total, 694 genes were down regulated by TROJAN knockdown, and 51 genes were positively correlated with TROJAN (R > 0.2, *P* < 0.05) in both FUSCC and TCGA cohort, including eight cell cycle-related genes according to Kyoto Encyclopedia of Genes and Genomes (KEGG) pathway analysis (Fig. 5a). Among these genes (CCNA2, MCM7, CDC45, CDK2, MCM3, PCNA, BUB1 and MCM6), we were most interested in CDK2, which was reported to be activated in PD-resistant cells [16, 17]. We hypothesized that CDK2 is a key target gene of TROJAN. In MCF7 PDR cells, we observed significant decreased of PD IC50 after CDK2 knockdown (Additional files 5: Fig. S5a-b). We next validated that the mRNA expression level of CDK2 was increased by the knockdown of TROJAN and was decreased by the knockdown of NKRF (Fig. 5b). Importantly, the knockdown of both TROJAN and NKRF restored the expression of CDK2 (Fig. 5c). Based on the luciferase reporter assay, the promoter activity of CDK2 was inhibited by TROJAN knockdown, while the promoter activity was activated by NKRF knockdown (Fig. 5d). Importantly, the CDK2 level and the total amount of CDK Thr160 phosphorylation level decreased upon TROJAN knockdown (Fig. 5e), indicating that CDK2 was inactivated. The expression of CDK2 was increased upon TROJAN overexpression (Fig. 5f). In a mammary fat pad xenograft model, we found that the expression of CDK2 was decreased in TROJAN knockdown tumors (Fig. 5g). Previous research has shown that NKRF has DNA-binding activity and represses the transcription of its target genes through interactions with other NF-κB factors at the promoter [15]. Through a public ChIP-Seq dataset (GSE31477), RELA could bind to the promoter of CDK2 (Fig. 5h) and overlapped with the H3K27ac and H3K4me3 signal (Additional files 5: Fig. S5c). We inferred that TROJAN and NKRF could regulate the promoter activity of CDK2 and could alter the expression of CDK2 at the transcriptional level. Using a ChIP assay, we observed a significant increase in the NKRF binding capacity at the CDK2 promoter after TROJAN knockdown (Fig. 5i). Similar to previous studies, we observed that PDR cells had sustained high levels of CDK2 Thr160 and RB1 S807/811 phosphorylation despite CDK4/6 inhibition (Fig. 5j) [16, 17]. Furthermore, the knockdown of TROJAN in PDR cells abrogated the phosphorylation of CDK2 and RB1 (Fig. 5k). We observed that the overexpression of CDK2 in TROJAN knockdown MCF7 cells could partially revert the proliferative inhibition compared to TROJAN knock down cells. However, the subsequent CDK2 silencing in TROJAN knockdown MCF7 cells did not affect proliferation (Fig. 5l). Taken together, these data indicate that TROJAN prevents NKRF binding at the CDK2 promoter and can regulate the expression level of CDK2 at the transcriptional level.

### TROJAN is positively correlated with CDK2 and MKI67 in clinical samples

**Fig. 6:**
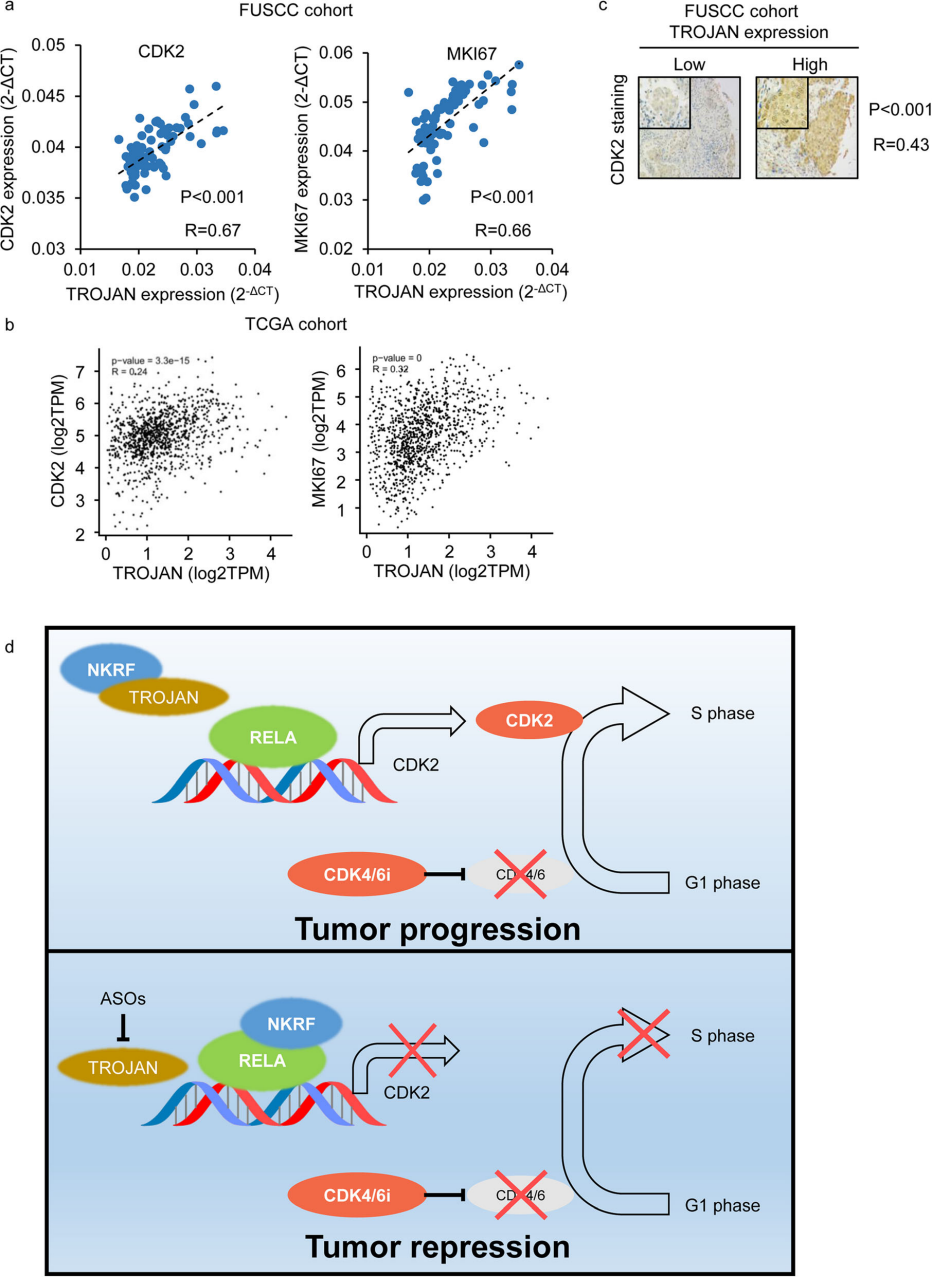
Correlation among TROJAN, CDK2 and MKI67 in clinical samples. **a** Correlation between TROJAN and CDK2/MKI67 expression, as detected by qRT-PCR in the FUSCC cohort. The correlations were determined by Pearson correlation analysis. **b** Correlation between TROJAN (ENST00000624228.1 as a substitute transcript) and CDK2/MKI67 expression in the TCGA cohort. The correlations were determined by Pearson correlation analysis. **c** Immunohistochemical detection of CDK2 in the FUSCC cohort. The correlations were determined by Pearson correlation analysis. Scale bar: 100 μm. **d** Schematic diagram of TROJAN regulation of the resistance of ER+ breast cancer to a CDK4/6 inhibitor via NKRF

To investigate the clinical correlation among TROJAN, CDK2 and the proliferation marker MKI67, we performed qRT-PCR to measure their expression in ER+ breast cancer specimens. We found that TROJAN was positively correlated with CDK2 and MKI67 at both the mRNA and protein levels (Fig. 6a). Similar results were also found by analyzing the TCGA cohort (Fig. 6b). Furthermore, TROJAN was also positively correlated with the CDK2 protein level, as detected by IHC (Fig. 6c).

## Discussion

In our current study, we report that the inhibition of TROJAN can suppress the expression of CDK2, blocking noncanonical CDK2-mediated S phase entry, and thus enhancing the efficacy of a CDK4/6 inhibitor (Fig. 6d). In this mechanism, TROJAN can bind to NKRF, an NF-κB pathway repressor, hindering the binding between NKRF with RELA and the CDK2 promoter. The inhibition of TROJAN can further impair the activation of CDK2.

In ER+ breast cancer, the CDK4/6-CCND1-RB1 axis is a critical pathway in tumor cell cycle regulation [23, 24]. Despite the impressive results of randomized trials with PD in ER+ breast cancer, some patients do not respond to this therapy [10–13]. Efforts are being made to identify markers that predict sensitivity to CD4/6 inhibition or that overcome resistance to CDK4/6 inhibition [25]. Formisano et al. found that FGFR1 amplification is a mechanism of CDK4/6 inhibitor resistance [8]. Higher FGFR1 expression was related to a worse response of CDK4/6 inhibition. The addition of an FGFR tyrosine kinase inhibitor could abrogate this resistance. Li et al. demonstrated that the inactivation of FAT1 promoted CDK4/6 inhibitor resistance [26]. The loss of FAT1 could cause Hippo pathway suppression, thus leading to CDK6-mediated CDK4/6 inhibitor resistance. Additionally, Herrera-Abreu et al. reported that the activation of CDK2 was an important canonical bypass mechanism of cell cycle progression during CDK4/6 inhibition [16]. Novel CDK4/6 inhibitor combinations, such as PI3K inhibitors and immune checkpoint inhibitors, are being explored [27, 28]. However, the link between lncRNA and CDK4/6 inhibition was poorly understood and led us to investigate the role of lncRNA in this process. Therefore, we selected one lncRNA, TROJAN, as a candidate. We previously proved that TROJAN triggers TNBC progression.

In our two previous studies, we showed that the lncRNA TROJAN (also named AK124454 or ENST00000624228.1), whose sequence is similar to human endogenous retrovirus long terminal repeat 70, was a biomarker predicting worse survival that promoted TNBC progression through the degradation of ZMYND8 [19, 29]. Additionally, the anti-TROJAN ASO we identified decreased TROJAN expression as well as impaired the proliferation and metastatic ability of TNBC in vivo. As measured by biochemical parameters, such as alanine aminotransferase, aspartate aminotransferase, total bilirubin and blood urea in ASO-treated mice, ASO therapy was proven to have limited toxicity. In this report, we established that TROJAN is highly expressed in ER+ breast cancer tissues and predicts poor survival. The inhibition of TROJAN impaired proliferation through cell cycle G1/S phase arrest. These results led us to investigate the effectiveness of a combination therapy with an anti-TROJAN ASO and a CDK4/6 inhibitor. Importantly, the in vitro and in vivo assays showed that the anti-TROJAN ASO combined with PD significantly suppressed tumor growth compared with that of either single agent. Two previous studies showed that a potential molecular mechanism of resistance to CDK4/6 inhibitors was the noncanonical activation of CDK2 [16, 17]. The efficacy of CDK2 inhibitors was not clear [30]. One pan-CDK inhibitor, dinaciclib (targeting CDK2, CDK5, CDK1 and CDK9), was still in phase 1 clinical trials (NCT03484520, NCT03484520, NCT01676753, NCT01676753, NCT02684617 and NCT02684617) [31]. Therefore, novel CDK2 inhibitors still need to be developed. Here, our research showed that the inhibition of TROJAN decreased the expression of CDK2 as well as its phosphorylation, and the inactivation of CDK2 mediated cell cycle entry. Our findings support the use of a strategy that inhibits CDK2/4/6 to overcome resistance to CDK4/6 inhibition. These data suggested that the anti-TROJAN ASO with PD might be a promising novel combination for treating ER+ breast cancer patients. Clearly, the widespread molecular profiling of patients receiving CDK4/6 inhibitors will be required to validate our results.

According to previous studies, lncRNAs could be involved in different biological processes in different tumor types [32]. For example, the lncRNA BCAR4 promoted the metastasis of TNBC and activated the sonic hedgehog pathway by binding to SNIP1 and PNUTS [33]. In ER+ breast cancer, BCAR4 promoted tumor proliferation and endocrine therapy resistance [34]. Several other lncRNAs, such as MALAT1 and HOTAIR [35–37], also participated in diverse pathways in different cancer types. The lncRNA MALAT1 served as a competitive endogenous RNA, sponging miRNAs, including miR-202 [38] (in gastric cancer) and miR-200c [39] (in endometrioid endometrial carcinoma). MALAT1 also bound to SUZ12, thus promoting bladder cancer metastasis [40]. In renal cell carcinoma, MALAT1 promoted tumor cell proliferation and invasion by interacting with EZH2 and miR-205 [41]. Interestingly, the transcriptome analysis during TROJAN knockdown showed distinct GO pathways between TNBC (pathways in cancer and focal adhesion) and non-TNBC cells (cell cycle and proliferation), suggesting that the biological function of TROJAN might be different in ER+ breast cancer and in TNBC. In addition, we found that NKRF was a key interacting protein with TROJAN in ER+ breast cancer. According to previous research, NKRF can inhibit tumor growth [14]. NKRF is widely known as a suppressor of the NF-κB pathway. NKRF can bind to the promoter of its target genes and abolish their transcriptional activity through the interaction of NF-κB factors, such as NFKB1, NFKB2 and RELA [15]. Intriguingly, our functional assay revealed that NKRF could at least partially rescue the proliferative inhibition caused by TROJAN knockdown, while ZMYND8 (a key TROJAN-interacting protein in TNBC) could barely reverse this effect. Thus, we conclude that TROJAN could be involved in different pathways in TNBC and ER+ breast cancer.

## Conclusions

In summary, our data suggest that combining an anti-TROJAN ASO with a CDK4/6 inhibitor is a promising combination of anticancer therapy. The potential mechanism of the TROJAN-NKRF-CDK2 regulatory pattern could support our findings.

## Supplementary information

**Additional files 1: Supplementary Figure 1.** TROJAN was highly expressed in tumor cells. (**a**) The expression of TROJAN in 16 paired ER+ breast cancer tissues and adjacent normal tissues. Paired t test. (**b**) RNA-seq of 53 tissues/8555 samples/570 donors from GTEx database (https://www.gtexportal.org/). Median transcript per million (TPM) of TROJAN, several other oncogenic lncRNAs as well as GAPDH were shown. (**b**) qRT-PCR detection of TROJAN expression in different cell lines.

**Additional files 2: Supplementary Figure 2**. The validation of CDK4/6i resistance cell lines. (**a**) IC50 values of palbociclib, ribociclib and abemaciclib in MCF7 and MCF7 palbociclib resistance cells (PDR). (**b**) Western blot images of MCF7 and PDR cells treated for 24 h with 0.1 μM palbociclib, ribociclib or abemaciclib and blotted for phospho-RB1 (p-RB1) S807/811, and total RB1. Unpaired t test, **p < 0.01 and ***p < 0.001. NS, not significant.

**Additional files 3: Supplementary Figure 3**. TROJAN regulates G1/S cell cycle pathway. (**a**) Pathway analysis of 1616 up regulated genes after TROJAN knockdown identified by a microarray. Top 10 pathways according to –log10 (*P* value) are shown. (**b**) Western blot images of Cyclin E1/2, Cyclin D1/2, p21 and p27 in MCF7 cells expressing TROJAN shRNAs. *n* = 3 independent experiments.

**Additional files 4: Supplementary Figure 4**. The validation of TROJAN interaction proteins in ER+ breast cancer. (**a**) Schematic diagram of the top four potential TROJAN-interacting proteins, as identified by mass spectrometry according to the intensity observed by mass spectrometry. (**b**) Western blot images of NKRF during NKRF knockdown. (**c**) In vitro growth curves of MCF7 cells expressing control (Ctrl) or NKRF shRNA. (**d**) Western blot images of ZMYND8 during ZMYND8 knockdown. (**e**) In vitro growth curves of MCF7 cells expressing TROJAN ± ZMYND8 shRNA, individually or in combination. Two-way ANOVA analysis, *p < 0.05 and ***p < 0.001. NS, not significant.

**Additional files 5: Supplementary Figure 5.** TROJAN regulates the transcriptional level of CDK2. (a) Western blot images of CDK2 during CDK2 knockdown or overexpression. (b) IC50 values of MCF7, MCF7 palbociclib resistance cells (PDR) and PDR ± CDK2 knockdown. Two-way ANOVA analysis was used. (c) ChIP-Seq signals of RELA, H3K27ac and H3K4me3 in lymphocyte at CDK2 nearby genomic location (GSE31477). **p < 0.01; NS, not significant.

## Data Availability

All data needed to evaluate the conclusions of this paper are presented in the paper and/or the supplementary materials. Additional data related to this paper may be requested from the corresponding author: Y.-Z.J. (yizhoujiang@fudan.edu.cn).

## Abbreviations

ASO: Antisense oligonucleotide
CDK: Cyclin-dependent kinase
ChIP: Chromatin immunoprecipitation
ER: Estrogen receptor
GO: Gene Ontology
IgG: Immunoglobulin G
IHC: Immunohistochemistry
ISH: In situ hybridization
KEGG: Kyoto Encyclopedia of Genes and Genomes
lncRNA: Long noncoding RNA
PD: Palbociclib
PDR: Resistance to palbociclib
qRT-PCR: Quantitative reverse transcription PCR
RFS: Relapse-free survival
RIP: RNA immunoprecipitation
SILAC: Stable isotope labeling with amino acids in cell culture
TCGA: The Cancer Genome Atlas
TNBC: Triple-negative breast cancer
