# Supplementary material for: LncRNA TROJAN promotes proliferation and resistance to CDK4/6 inhibitor via CDK2 transcriptional activation in ER+ breast cancer
Source: Mol Cancer. 2020 May 11;19:87. doi: 10.1186/s12943-020-01210-9 (PMC7212688; doi:10.1186/s12943-020-01210-9)
Supplement: Supplementary file 1 — Additional files 1: Supplementary Figure 1. TROJAN was highly expressed in tumor cells. (a) The expression of TROJAN in 16 paired ER+ breast cancer tissues and adjacent normal tissues. Paired t test. (b) RNA-seq of 53 tissues/8555 samples/570 donors from GTEx database (https://www.gtexportal.org/). Median transcript per million (TPM) of TROJAN, several other oncogenic lncRNAs as well as GAPDH were shown. (b) qRT-PCR detection of TROJAN expression in different cell lines. [file 12943_2020_1210_MOESM1_ESM.pdf]

Additional files 1: Supplementary Figure 1. TROJAN was highly expressed in tumor cells.

(**a**) The expression of TROJAN in 16 paired ER+ breast cancer tissues and adjacent normal tissues. Paired t test.

(**b**) RNA-seq of 53 tissues/8555 samples/570 donors from GTEx database (https://www.gtexportal.org/). Median transcript per million (TPM) of TROJAN, several other oncogenic lncRNAs as well as GAPDH were shown.

(**b**) qRT-PCR detection of TROJAN expression in different cell lines.

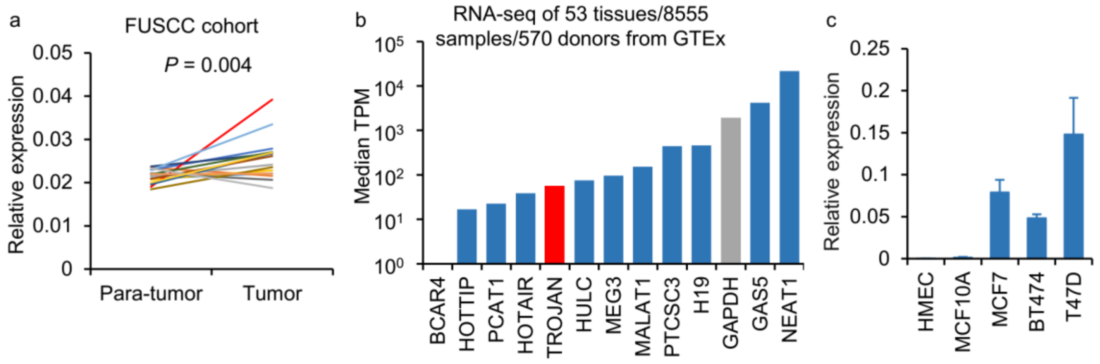

Figure S1
