# Supplementary material for: LncRNA TROJAN promotes proliferation and resistance to CDK4/6 inhibitor via CDK2 transcriptional activation in ER+ breast cancer
Source: Mol Cancer. 2020 May 11;19:87. doi: 10.1186/s12943-020-01210-9 (PMC7212688; doi:10.1186/s12943-020-01210-9)
Supplement: Supplementary file 2 — Additional files 2: Supplementary Figure 2. The validation of CDK4/6i resistance cell lines. (a) IC50 values of palbociclib, ribociclib and abemaciclib in MCF7 and MCF7 palbociclib resistance cells (PDR). (b) Western blot images of MCF7 and PDR cells treated for 24 h with 0.1 μM palbociclib, ribociclib or abemaciclib and blotted for phospho-RB1 (p-RB1) S807/811, and total RB1. Unpaired t test, **p < 0.01 and ***p < 0.001. NS, not significant. [file 12943_2020_1210_MOESM2_ESM.pdf]

Additional files 2: Supplementary Figure 2. The validation of CDK4/6i resistance cell lines.

(**a**) IC50 values of palbociclib, ribociclib and abemaciclib in MCF7 and MCF7 palbociclib resistance cells (PDR).

(**b**) Western blot images of MCF7 and PDR cells treated for 24 hours with 0.1 μM palbociclib, ribociclib or abemaciclib and blotted for phospho-RB1 (p-RB1) S807/811, and total RB1.

Unpaired t test, **p < 0.01 and ***p < 0.001. NS, not significant.

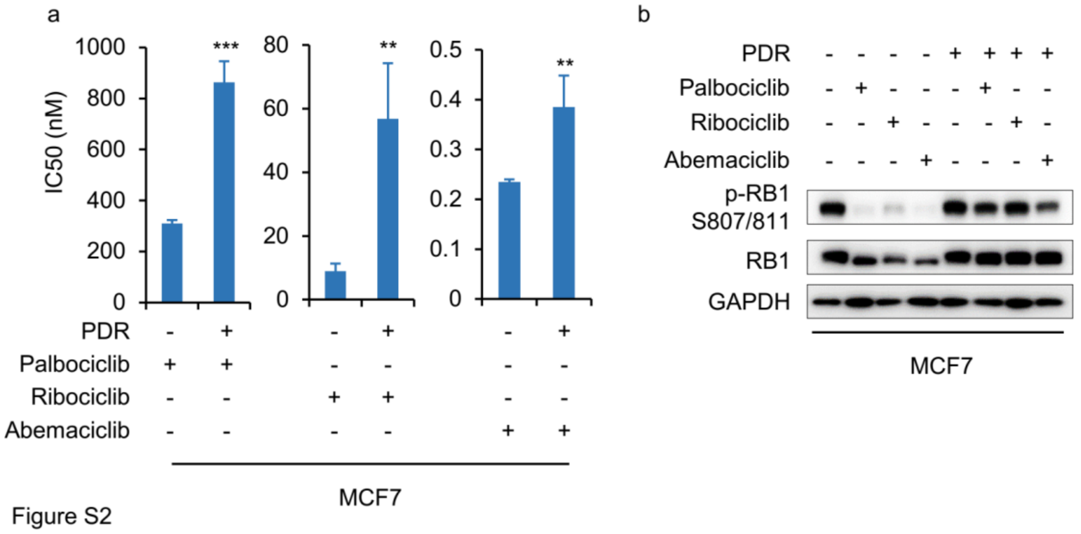

Figure S2
