# Supplementary material for: LncRNA TROJAN promotes proliferation and resistance to CDK4/6 inhibitor via CDK2 transcriptional activation in ER+ breast cancer
Source: Mol Cancer. 2020 May 11;19:87. doi: 10.1186/s12943-020-01210-9 (PMC7212688; doi:10.1186/s12943-020-01210-9)

Additional files 3: Supplementary Figure 3. TROJAN regulates G1/S cell cycle pathway.

(**a**) Pathway analysis of 1616 up regulated genes after TROJAN knockdown identified by a microarray. Top 10 pathways according to –log10 (P value) are shown.

(**b**) Western blot images of Cyclin E1/2, Cyclin D1/2, p21 and p27 in MCF7 cells expressing TROJAN shRNAs. n = 3 independent experiments.

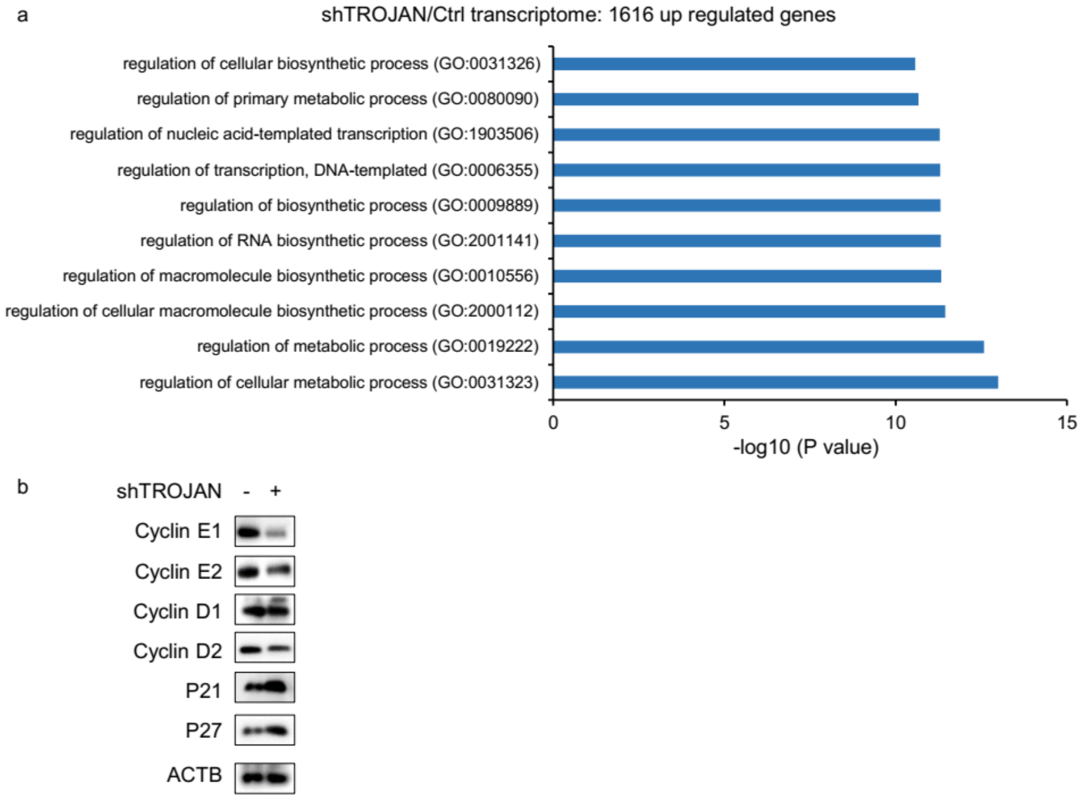

Supplement: Supplementary file 3 — Additional files 3: Supplementary Figure 3. TROJAN regulates G1/S cell cycle pathway. (a) Pathway analysis of 1616 up regulated genes after TROJAN knockdown identified by a microarray. Top 10 pathways according to –log10 (P value) are shown. (b) Western blot images of Cyclin E1/2, Cyclin D1/2, p21 and p27 in MCF7 cells expressing TROJAN shRNAs. n = 3 independent experiments. [file 12943_2020_1210_MOESM3_ESM.pdf]
