# Supplementary material for: LncRNA TROJAN promotes proliferation and resistance to CDK4/6 inhibitor via CDK2 transcriptional activation in ER+ breast cancer
Source: Mol Cancer. 2020 May 11;19:87. doi: 10.1186/s12943-020-01210-9 (PMC7212688; doi:10.1186/s12943-020-01210-9)
Supplement: Supplementary file 4 — Additional files 4: Supplementary Figure 4. The validation of TROJAN interaction proteins in ER+ breast cancer. (a) Schematic diagram of the top four potential TROJAN-interacting proteins, as identified by mass spectrometry according to the intensity observed by mass spectrometry. (b) Western blot images of NKRF during NKRF knockdown. (c) In vitro growth curves of MCF7 cells expressing control (Ctrl) or NKRF shRNA. (d) Western blot images of ZMYND8 during ZMYND8 knockdown. (e) In vitro growth curves of MCF7 cells expressing TROJAN ± ZMYND8 shRNA, individually or in combination. Two-way ANOVA analysis, *p < 0.05 and ***p < 0.001. NS, not significant. [file 12943_2020_1210_MOESM4_ESM.pdf]

Additional files 4: Supplementary Figure 4. The validation of TROJAN interaction proteins in ER+ breast cancer.

(**a**) Schematic diagram of the top four potential TROJAN-interacting proteins, as identified by mass spectrometry according to the intensity observed by mass spectrometry.

(**b**) Western blot images of NKRF during NKRF knockdown.

(**c**) In vitro growth curves of MCF7 cells expressing control (Ctrl) or NKRF shRNA.

(**d**) Western blot images of ZMYND8 during ZMYND8 knockdown.

(**e**) In vitro growth curves of MCF7 cells expressing TROJAN ± ZMYND8 shRNA, individually or in combination.

Two-way ANOVA analysis, *p < 0.05 and ***p < 0.001. NS, not significant.

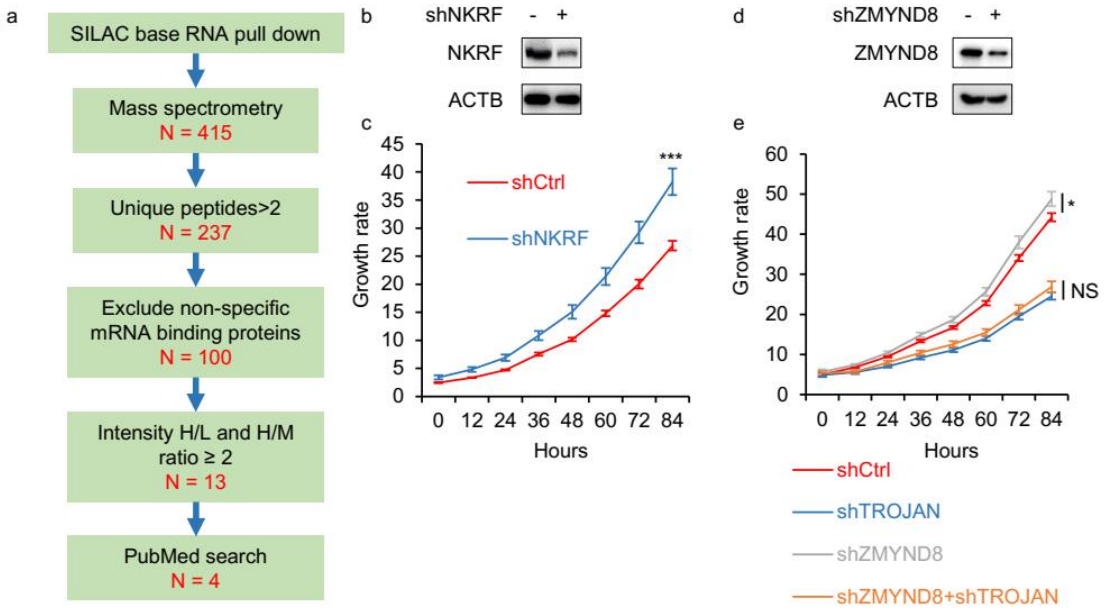

Figure S4
