# Supplementary material for: LncRNA TROJAN promotes proliferation and resistance to CDK4/6 inhibitor via CDK2 transcriptional activation in ER+ breast cancer
Source: Mol Cancer. 2020 May 11;19:87. doi: 10.1186/s12943-020-01210-9 (PMC7212688; doi:10.1186/s12943-020-01210-9)
Supplement: Supplementary file 5 — Additional files 5: Supplementary Figure 5. TROJAN regulates the transcriptional level of CDK2. (a) Western blot images of CDK2 during CDK2 knockdown or overexpression. (b) IC50 values of MCF7, MCF7 palbociclib resistance cells (PDR) and PDR ± CDK2 knockdown. Two-way ANOVA analysis was used. (c) ChIP-Seq signals of RELA, H3K27ac and H3K4me3 in lymphocyte at CDK2 nearby genomic location (GSE31477). **p < 0.01; NS, not significant. [file 12943_2020_1210_MOESM5_ESM.pdf]

Additional files 5: Supplementary Figure 5. TROJAN regulates the transcriptional level of CDK2.

(a) Western blot images of CDK2 during CDK2 knockdown or overexpression.

(b) IC50 values of MCF7, MCF7 palbociclib resistance cells (PDR) and PDR ± CDK2 knockdown. Two-way ANOVA analysis was used.

(c) ChIP-Seq signals of RELA, H3K27ac and H3K4me3 in lymphocyte at CDK2 nearby genomic location (GSE31477).

**p < 0.01; NS, not significant.

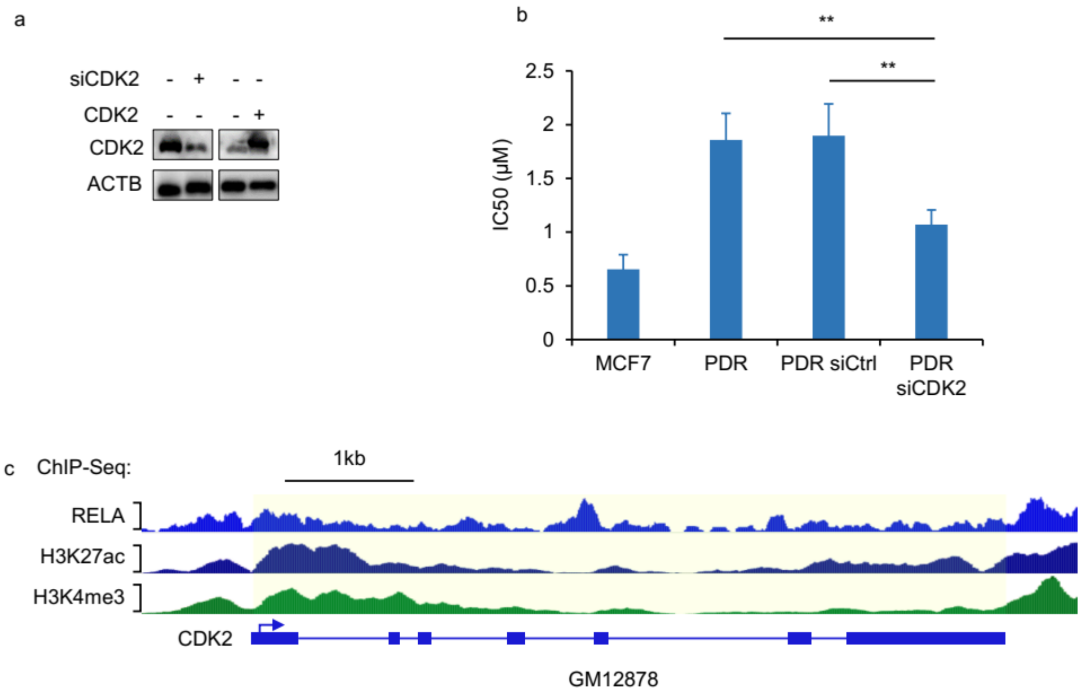

Figure S5
